# Hepatic Steatosis as a Marker of Metabolic Dysfunction

**DOI:** 10.3390/nu7064995

**Published:** 2015-06-19

**Authors:** Elisa Fabbrini, Faidon Magkos

**Affiliations:** Center for Human Nutrition and Atkins Center of Excellence in Obesity Medicine, Washington University School of Medicine, St. Louis, MO 63110, USA; E-Mail: efabbrini@dom.wustl.edu

**Keywords:** NAFLD, nonalcoholic fatty liver disease, liver steatosis, glucose metabolism, lipid metabolism, insulin resistance, obesity, fatty acid metabolism, lipolysis, VLDL secretion

## Abstract

Nonalcoholic fatty liver disease (NAFLD) is the liver manifestation of the complex metabolic derangements associated with obesity. NAFLD is characterized by excessive deposition of fat in the liver (steatosis) and develops when hepatic fatty acid availability from plasma and *de novo* synthesis exceeds hepatic fatty acid disposal by oxidation and triglyceride export. Hepatic steatosis is therefore the biochemical result of an imbalance between complex pathways of lipid metabolism, and is associated with an array of adverse changes in glucose, fatty acid, and lipoprotein metabolism across all tissues of the body. Intrahepatic triglyceride (IHTG) content is therefore a very good marker (and in some cases may be the cause) of the presence and the degree of multiple-organ metabolic dysfunction. These metabolic abnormalities are likely responsible for many cardiometabolic risk factors associated with NAFLD, such as insulin resistance, type 2 diabetes mellitus, and dyslipidemia. Understanding the factors involved in the pathogenesis and pathophysiology of NAFLD will lead to a better understanding of the mechanisms responsible for the metabolic complications of obesity, and hopefully to the discovery of novel effective treatments for their reversal.

## 1. Introduction

Obesity is associated with altered physiological functions in almost all tissues and organ systems of the body. The liver in obese people is characterized by an accumulation of intrahepatic triglyceride (IHTG), *i.e.*, steatosis, which is the hallmark feature of nonalcoholic fatty liver disease (NAFLD). This can progress to nonalcoholic steatohepatitis (NASH) if inflammation is also present, with or without fibrosis, and can ultimately lead to cirrhosis. Inflammatory markers such as circulating interleukin (IL)-6, therefore, can discriminate NAFLD from NASH with high specificity [1]. Due to the increased incidence of obesity worldwide, NAFLD has become an important public health problem because of its high prevalence, potential progression to severe liver disease, and strong link with important cardiometabolic risk factors [2]. NAFLD is associated with increased risk for developing insulin resistance, dyslipidemia (high plasma triglyceride and/or low high density lipoprotein-cholesterol concentrations), and hypertension [3], and is an independent predictor of the development of pre-diabetes and type 2 diabetes [4,5,6]. In this review we provide a concise yet comprehensive assessment of the complex physiological interactions that adversely affect metabolic function in obesity-related NAFLD.

## 2. Diagnosis and Prevalence of NAFLD

The defining characteristic of NAFLD is the presence of an increased content of triglyceride within the cytoplasm of hepatocytes, in the absence of excessive alcohol consumption (defined as >20 g/day for men and >10 g/day for women) or other causes of fat deposition in the liver (certain medications, autoimmune or genetic diseases, and viral infections). Excessive hepatic lipid content (*i.e.*, steatosis) has been traditionally assessed by means of a liver biopsy and defined by chemical means, when IHTG content exceeds 5% of liver volume or liver weight [7], or by histological means, when 5% of hepatocytes contain visible intracellular triglyceride [8,9]. In the past decade, advances in imaging technology allowed the noninvasive evaluation of IHTG content by magnetic resonance spectroscopy (MRS) in large numbers of subjects [10,11]. Based on MRS measurement of liver fat in a group of subjects considered to be at low-risk for NAFLD (*i.e.*, subjects of normal weight, with normal fasting serum glucose and alanine aminotransferase concentrations, and without diabetes mellitus), the upper “normal” limit of IHTG has been defined as 5.6% of liver volume, which represented the 95th percentile in this reference population [10]. In most clinical research studies, NAFLD is therefore defined as IHTG >10% of liver volume. It is important to point out that none of the values proposed for the diagnosis of NAFLD are based on the relationship between IHTG and either metabolic or clinical outcomes. In fact, data suggest that the relationship between metabolic function and IHTG content in obese subjects is linear, without evidence of an obvious threshold that can be used to define normality [12].

The prevalence rate of NAFLD increases with increasing body mass index (BMI) [13], and is influenced by age, sex, racial/ethnic background and genetic variation [14,15,16]. Overall, it is estimated that ~30% of the adult and ~15% of the pediatric populations in the United States have NAFLD, with a prevalence rate that reaches ~90% in the severely obese population [17]. The prevalence of NAFLD is also extremely high in overweight/obese patients with type 2 diabetes (~70%) [18,19,20] even in the presence of normal levels of aminotransferases [20].

## 3. Metabolic Dysfunction in NAFLD

The liver serves as an intermediary organ between exogenous (*i.e.*, dietary) and endogenous sources of energy and the various extrahepatic organs that consume energy [21], and performs a vast number of essential biochemical functions, either continuously or in biological rhythms (e.g., circadian), or according to specific states (e.g., fasting and feeding) and tissue requirements [22,23], which are necessary for whole-body metabolic homeostasis [23]. For instance, the liver maintains plasma glucose concentration within a tightly controlled narrow range, by releasing glucose into the bloodstream in the postabsorptive state and taking it up in the postprandial state. The liver is also central in normal lipid and lipoprotein metabolism, by taking up and synthesizing fatty acids, channeling them towards oxidative or esterification pathways, and secreting them in the form of triglyceride in the core of very low density lipoproteins (VLDL). NAFLD has been associated with alterations in most of the liver’s physiological functions.

## 4. Glucose Metabolism

During the postabsorptive state, when no exogenous carbohydrate is available, endogenous glucose production (>90% of which comes from the liver) increases to maintain plasma glucose concentration [24]. The rise in endogenous glucose production during fasting is the result of accelerated gluconeogenesis (the formation of glucose from non-glucose precursors such as lactate, pyruvate, glucogenic amino acids and glycerol) and glycogenolysis (the degradation of glycogen to glucose units), concomitant to the low fasting plasma insulin concentration and/or the low insulin/glucagon ratio [24,25]. In healthy subjects, hepatic gluconeogenesis and glycogenolysis contribute approximately equally to basal endogenous glucose production [25,26]. After a mixed meal, endogenous glucose production decreases as a result of the increasing insulin concentration and insulin/glucagon ratio [24]. Hepatic glucose production is very sensitive to changes in prevailing insulinemia: an increase in insulin concentration from 11 mU/L (a typical fasting insulin concentration in non-diabetic obese subjects) to only 37 mU/L causes a 70% decline in hepatic glucose production, whereas at a plasma insulin concentration of 53 mU/L, hepatic glucose production is suppressed by almost 90% [27]. Insulin inhibits hepatic glucose production predominantly by stimulating glycogen synthesis (direct pathway) and, to a lesser extent, by inhibiting proteolysis and lipolysis, thereby limiting the supply of gluconeogenic precursors for synthesis of new glucose (indirect pathway) [25].

### 4.1. Hepatic Insulin Action in NAFLD

Many studies have found that hepatic insulin resistance, *i.e.*, the diminished ability of circulating insulin to suppress hepatic glucose production, is directly related to IHTG content [12,28,29,30,31], independent of age, sex, BMI, waist circumference, percent body fat, and visceral fat mass [12,28,30,31,32,33]. For example, individuals with NAFLD have 20% to 35% lower suppression of endogenous glucose production in response to insulin infusion compared to BMI- and body fat- matched subjects [28,34]. A recent study found that men with high IHTG content have higher flux rates through all three pathways contributing to glucose production during hyperinsulinemia (gluconeogenesis from tricarboxylic acid cycle intermediates; gluconeogenesis from glycerol, and glycogenolysis) compared to those with low IHTG, but no differences during fasting. Consequently, increased production of glucose during hyperinsulinemia in NAFLD results from inadequate suppression of all the supporting fluxes of glucose production, rather than from a selective dysregulation of individual glucose production pathways [35].

#### Molecular Mechanisms Linking Hepatic Steatosis to Insulin Resistance

Even though the link between hepatic insulin resistance and NAFLD is well-established, it is still not known whether NAFLD is the cause, or the consequence, of insulin resistance, or possibly both. Studies conducted in rodents have found only a few days of high-fat feeding are sufficient to induce liver steatosis and hepatic insulin resistance, before any change in systemic metabolic function and inflammation occurs before the development of obesity [36,37]. Experimental treatments such as high fat feeding or bolus lipid administration promote excessive accumulation of intracellular lipid intermediates generated by fatty acid metabolism, particularly diacylglycerol (DAG) species, which provide a putative causal link between increased IHTG content and hepatic insulin resistance. DAG accumulation in the liver has been shown to activate classical (β) and novel (δ and ε) protein kinase C isoforms [38,39,40,41], which can in fact interfere with insulin action by disrupting normal insulin receptor function, ultimately leading to impaired insulin-mediated suppression of hepatic glucose production [42]. Recent studies in genetically modified rodents show that PKCβ, PKCδ and PKCε are all independent regulators of hepatic lipogenic genes and of IHTG accumulation, and that mice lacking any of these PKC isoforms are protected from high-fat diet induced insulin resistance at the liver but also at the whole body level [37,39,40,41,43,44]. In obese human subjects, intrahepatic DAG content, but not ceramides or acylcarnitines (other derivatives of fatty acid metabolism), is inversely correlated with the ability of insulin to suppress endogenous glucose production [45]. Similarly, intrahepatic DAG content correlates with the HOmeostasis Model Assessment (HOMA) score [46], which is a crude index of hepatic but also muscle insulin resistance [47]. Monoacylglycerol acyltransferase (MGAT) enzymes, which convert monoacylglycerol to DAG, have recently been considered of potential relevance to the mechanisms of obesity-related hepatic steatosis because: (i) the product of MGAT activity (DAG) is a key mediator of insulin resistance in several tissues (*see above*), (ii) gene expression of the MGAT enzymes is induced in the steatotic liver of both rodents [48] and humans [49], and (iii) weight loss in obese subjects causes a reduction in MGAT gene expression concomitant to an improvement in insulin sensitivity and resolution of hepatic steatosis [49]. Moreover, a recent study in diet-induced obese mice found that inhibition of MGAT activity in the liver leads to an improvement in glucose tolerance and in hepatic insulin signaling, suggesting that modulating MGAT activity could represent a possible target to alleviate obesity-associated hepatic metabolic dysfunction [50].

The endocannabinoid system has also been involved in the development of intrahepatic lipid accumulation and hepatic insulin resistance. First of all, specific endocannabinoids as well as endocannabinoid receptors are increased in the liver of mouse models of diet-induced obesity [51]. Moreover, activation of cannabinoid 1 receptor in the liver promotes transcription factors that can induce *de novo* lipogenesis and activation of the phosphatidic acid phosphatase, lipin-1 [52]. This can increase the formation of DAG which can in turn inhibit hepatic insulin action [52]. The endoplasmic reticulum (ER) stress response has recently been proposed to play a crucial role in the development and progression of hepatic steatosis, as well as in the pathogenesis of hepatic insulin resistance associated with NAFLD [53]. Activation of the ER-stress response can induce hepatic steatosis by activating lipogenic pathways through stimulation of several genes involved in lipid synthesis [53]. Moreover, activation of the ER-stress response induced by an increase in liver saturated fatty acid content [54] can cause hepatic insulin resistance through activation of the c-Jun NH2-terminal kinase (JNK) pathway, which inhibits insulin signaling through inactivation and/or degradation of insulin receptor substrate (IRS) 1 [55]. The putative role of endoplasmic reticulum stress in mediating steatosis and hepatic insulin resistance is supported by the observations that ER-stress is increased in the liver of obese subjects with NAFLD [56], and decreases with weight loss, concomitant to an improvement in hepatic insulin sensitivity and the resolution of steatosis [57].

Intrahepatic inflammation may also provide a link between NAFLD and insulin resistance. Diet-induced and genetically-induced obesity in rodent models are associated with hepatic steatosis, insulin resistance, and increased hepatic nuclear factor (NF)-κB activity [36,58]. Activation of the NF-κB pathway in the liver causes hepatic inflammation, increases local and circulating interleukin-6 (IL-6), and results in both hepatic and skeletal muscle insulin resistance [58]. In addition, administration of an antibody neutralizing IL-6 to mice fed a high-fat diet upregulates skeletal muscle glucose transport and modulates production of adipose tissue adipokines, ultimately improving hepatic insulin resistance and steatosis [59]. These observations suggest that steatosis can activate intrahepatic inflammatory pathways, which upregulate the production of proinflammatory cytokines that can lead to both hepatic and peripheral insulin resistance. Nevertheless, systemic improvement of inflammatory status can ameliorate hepatic steatosis by modulating adipokine production and peripheral glucose metabolism. These findings underscore the complex metabolic interactions between the liver and extrahepatic tissues.

### 4.2. Muscle Insulin Action in NAFLD

**Figure 1:**
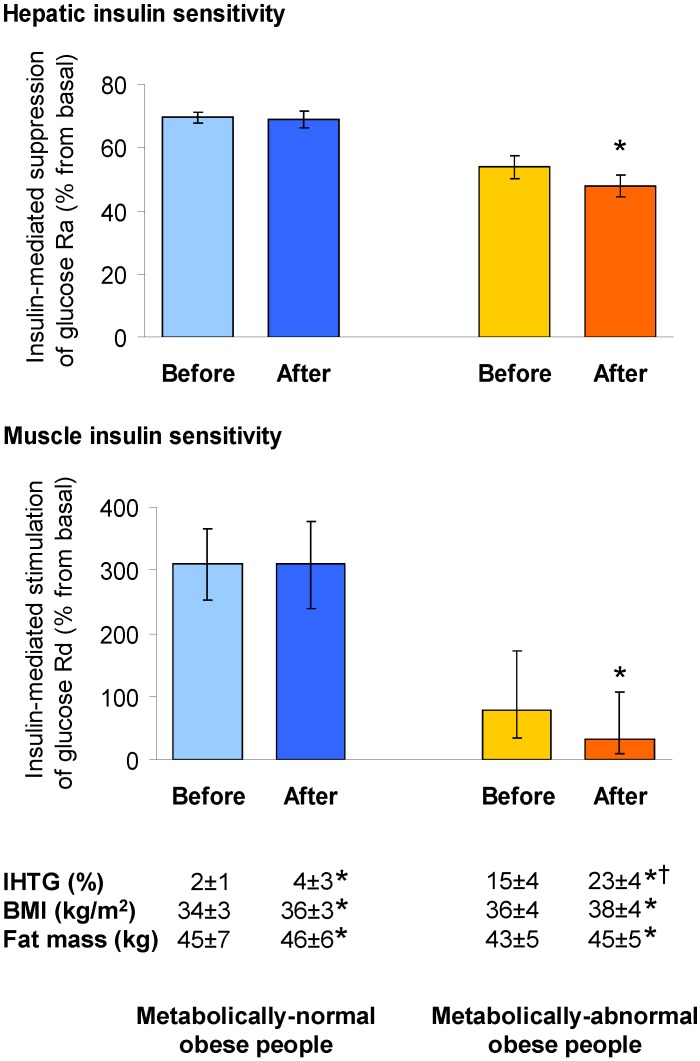
NAFLD as a marker of metabolic dysfunction. Metabolically-normal (*n* = 12) and metabolically-abnormal (*n* = 8) obese people, defined as having low or high intrahepatic triglyceride (IHTG) content, respectively, but matched on body mass index (BMI) and fat mass, were prescribed a hypercaloric diet for 7–8 weeks, until they gained ~6% of their initial body weight. Hepatic insulin sensitivity (*top panel*) and skeletal muscle insulin sensitivity (*bottom panel*), determined by using a two-stage hyperinsulinemic-euglycemic clamp, decreased after weight gain in metabolically-abnormal but not in metabolically-normal people, suggesting that low liver fat “protects” against the adverse metabolic effects of moderate weight gain. Drawn with data provided in reference [34]. Values for insulin-mediated suppression of glucose rate of appearance (Ra) are means ± SEMs, and those for insulin-mediated stimulation of glucose rate of disappearance (Rd) are back-log-tra nsformed means with 95% confidence intervals. Hepatic and muscle insulin sensitivity were greater and IHTG content was lower in metabolically-normal than in metabolically-abnormal people, both before and after weight gain, whereas BMI and fat mass were not different. * Value after weight gain is significantly different from corresponding value before weight gain. ^†^ Change induced by weight gain (in absolute terms) is greater in metabolically-abnormal than in metabolically-normal people.

Many studies in human subjects have found that NAFLD is associated not only with impaired hepatic insulin action, but also with profound insulin resistance in skeletal muscle, *i.e.*, with reduced ability of circulating insulin to stimulate muscle glucose uptake [12,28,29,30,32,34]. The presence of NAFLD is therefore a robust marker of multi-organ insulin resistance and metabolic dysfunction in obese people [29,32,34,60,61], and may also be a marker of the worsening of metabolic dysfunction in response to weight gain. A recent study demonstrated that obese people with normal IHTG content are “protected” from the development of adverse metabolic changes after moderate weight gain (~6% of initial body weight); conversely, BMI- and body fat mass-matched obese subjects with high IHTG content (*i.e.*, NAFLD) are prone to further deterioration of insulin action in the liver, adipose tissue, and skeletal muscle in response to moderate weight gain [34] (Figure 1). Results from this study demonstrate that IHTG content can be used to identify obese people who are prone to, or protected from, the development of metabolic disease, at least in response to short-term moderate weight gain. This suggests that NAFLD can be considered among the criteria to identify obese subjects at a higher risk of deterioration in metabolic function with additional weight gain, who are therefore in need of more aggressive weight management therapy. This can potentially have important implications for public health, not only because the natural history of obesity (*i.e.*, obese people typically gain more weight and become more obese over time [62]) but also because there is likely a limit of weight gain beyond which “metabolically-healthy” obese people are no longer protected from a deterioration in metabolic function [63].

### 4.3. β-Cell Dysfunction in NAFLD

Recent studies found that NAFLD is also associated with significant pancreatic β-cell dysfunction in non-diabetic obese subjects [64,65], but whether this is a primary defect or develops secondarily in response to insulin resistance is not clear. Individuals with NAFLD have an exaggerated β-cell insulin secretory response to an oral glucose load independent of BMI, age and sex; and a decline in β-cell index, which reflects pancreatic β-cell function in the setting of underlying insulin resistance. This likely reflects deterioration of insulin sensitivity, resulting in augmented insulin secretion to compensate for the impaired insulin action and maintain glucose homeostasis [65].

### 4.4. Dissociation between Insulin Resistance and NAFLD

Steatosis does not always coincide with insulin resistance. Studies in rodent models found that overexpression of hepatic DGAT [66], blockade of hepatic VLDL secretion [67], and pharmacological blockade of *beta*-oxidation [68] cause hepatic steatosis but do not impair insulin action; conversely, inhibition of IHTG synthesis ameliorates hepatic steatosis but does not improve insulin action [69]. Likewise, patients with familial hypobetalipoproteinemia develop hepatic steatosis because of a genetic deficiency of hepatic apolipoprotein B-100 synthesis, which results in virtually absent secretion of VLDL particles; however, these individuals do not develop hepatic or peripheral insulin resistance [70,71]. Therefore, IHTG accumulation does not necessarily cause insulin resistance. It is thus possible that esterification of surplus fatty acids to biologically inert triglyceride protects the hepatocyte from the potentially cytotoxic effects of excessive fatty acid availability [72,73,74]. Along these lines, IHTG accumulation may be secondary to a primary defect in skeletal muscle insulin action that diverts carbohydrate from muscle (for storage as muscle glycogen) to the liver (for *de novo* fatty acid synthesis) [75]. These findings underscore the complexity of the links between NAFLD and insulin action and suggest that the relationship between IHTG and multi-organ insulin resistance is likely multifactorial and certainly not unidirectional.

## 5. Lipid and Lipoprotein Metabolism

Fat accumulates within hepatocytes when the amount of fatty acids entering the liver (non-esterified fatty acids taken up from plasma and fatty acids synthesized *de novo* in hepatocytes) is greater than the amount of fatty acids exiting (fatty acid oxidation and triglyceride-bound fatty acid secretion) [76]. Therefore, intrahepatic triglyceride content is the net result of complex interactions among various metabolic pathways: (1) hepatic uptake of plasma free fatty acids (FFA), released mainly from lipolysis of adipose tissue triglycerides, (2) hepatic *de novo* lipogenesis, (3) hepatic fatty acid oxidation and ketogenesis, and (4) fatty acid secretion in VLDL-triglyceride.

### 5.1. Intrahepatic Fatty Acid Availability in NAFLD

#### 5.1.1. Hepatic Fatty Acid Uptake

Circulating, albumin-bound fatty acids are taken up by hepatocytes via both non-saturable simple diffusion and saturable facilitated transport. The total uptake of FFA by the liver directly depends on the concentration of FFA in plasma, and also on the capacity of hepatocytes for FFA uptake, which is predominantly related to the number and activity of fatty acid carrier and transport proteins [77]. Facilitated transport accounts for more than two-thirds of total hepatic fatty acid uptake under most circumstances and is mediated by specialized fatty acid carrier and transport proteins, *i.e.*, fatty acid binding protein (FABP), fatty acid translocase (FAT/CD36), and fatty acid transport polypeptide (FATP) [77,78], but also depends on the presence of scaffolding proteins such as caveolin-1 and lipolytic enzymes, such as phospholipase A2 [76,79]. In the postabsorptive state, the major source of fatty acids for the liver is the systemic plasma FFA pool, which consists of FFA derived predominantly from the lipolysis of subcutaneous adipose tissue triglycerides, and to a small extent from lipolysis of triglyceride in circulating lipoproteins. FFA from the systemic circulation reach the liver through the hepatic artery and the portal vein after passage through the splanchnic tissues. Although lipolysis of visceral adipose tissue triglycerides releases additional FFA directly into the portal vein, the relative contribution of visceral adipose tissue to the total hepatic FFA delivery through the portal vein is quite small, with only about 5% and 20% of portal vein FFA originating from visceral fat lipolysis in lean and obese subjects, respectively; the majority of portal vein FFA are derived from subcutaneous adipose tissue lipolysis after passage through the splanchnic tissues [80]. The modest contribution of visceral adipose tissue to hepatic FFA delivery casts doubt on its importance in metabolic dysfunction, and this notion is in line with findings on its contribution to circulating inflammatory markers as well [81].

The basal rate of fatty acid release in the systemic circulation increases directly with increasing body fat mass in both men and women [82], however, independent of the degree of obesity, the presence of NAFLD is typically associated with 35%–45% greater basal lipolytic rates [30,60,83,84] and with impaired insulin-mediated suppression of adipose tissue lipolysis [12,30,83]. These defects in the regulation of adipose tissue lipolysis are also evident in patients with NASH [85]. Therefore, for a given amount of body fat, the release of FFA into plasma is greater in patients with NAFLD than those without NAFLD during both fasting and feeding, *i.e.*, throughout the day [86]. In addition, gene expression of hepatic lipolytic enzymes, such as hepatic lipase and lipoprotein lipase, are greater in obese subjects with NAFLD than those without NAFLD [87,88,89]. These findings suggest that NAFLD is associated with greater delivery of circulating FFA to the liver. The capacity of the liver to take up fatty acids is also likely augmented in NAFLD, because of increased hepatic gene expression of several key proteins involved in lipid uptake and intracellular transport [89]. For instance, hepatic CD36 mRNA and protein levels are 65%–85% greater in subjects with NAFLD than in BMI-matched subjects without NAFLD [90], and expression of the FABP isoforms FABP-4 and FABP-5 in the liver correlates directly with IHTG content [88]. These results highlight the importance of dysregulation of hepatic fatty acid uptake at many levels, but in a coordinated manner, in the excessive accumulation of triglyceride in the liver. Nevertheless, not all studies find evidence of upregulated hepatic fatty acid uptake in NAFLD or NASH [91], suggesting that other factors, perhaps localized inside the liver, are also likely involved in the pathogenesis of hepatic steatosis.

#### 5.1.2. Hepatic *de Novo* Lipogenesis

The liver, much like adipose tissue, can synthesize fatty acids intracellularly from simple 2-carbon precursors (*i.e.*, acetyl-CoA). In a complex polymerization process taking place in the cytoplasm, acetyl-CoA carboxylase (ACC) catalyzes the irreversible carboxylation of acetyl-CoA to produce malonyl-CoA, which then undergoes several cycles of condensation, decarboxylation, and reduction reactions to form a 16-carbon saturated fatty acid (*i.e.*, palmitate) [92,93]. The synthesis of fatty acids is catalyzed by fatty acid synthase (FAS), which is a single, large, multifunctional polypeptide enzyme complex comprising seven distinct enzymatic activities. Regulation of *de novo* lipogenesis in the liver occurs: i) by the modulation of the amount and activity of various key enzymatic activities of the process, including FAS, ACC-1 and ACC-2, AMP-activated protein kinase, diacylglycerol acyltransferase (DGAT) 1 and 2, and stearoyl-CoA desaturase 1 (SCD1), ii) by the expression and activation state of nuclear transcription factors, including sterol regulatory element binding proteins (SREBPs), carbohydrate responsive element binding protein (ChREBP), liver X receptor α (LXRα), farnesoid X receptor (FXR), and peroxisome proliferator-activated receptors (PPARs), and iii) by the rate of delivery of acetyl-CoA to the cytoplasm [78,93]. Although several regulatory features of *de novo* lipogenesis in the liver are similar to those in adipose tissue [92], there is tissue-specific regulation which is likely critical for the metabolic dysfunction in obesity and NAFLD [94].

In non-obese healthy subjects, hepatic *de novo* lipogenesis in the fasting and postprandial states typically accounts for ≤5% and ≤10% of all fatty acids incorporated in IHTG and VLDL-triglyceride, respectively [95,96], but its contribution can be as much as 3–5 times greater in patients with NAFLD, accounting for 15% to 25% of all fatty acids in IHTG and VLDL-triglyceride in the postabsorptive state [95,97,98]. These results are consistent with the reported upregulation of gene expression of several of the enzymes involved in hepatic *de novo* fatty acid synthesis in NAFLD [99,100,101]. Although the absolute rates of hepatic *de novo* lipogenesis in the fasting state are quite small, and thus unlikely to be primarily responsible for excessive fat accumulation in the liver, these rates increase temporally in the postprandial state [102] and abnormally large increases in *de novo* lipogenesis following meal ingestion may precede the excessive fat accumulation in the liver (*i.e.*, NAFLD) [75]. Specific dietary habits have been implicated in the augmentation of hepatic de novo lipogenesis and the development of NAFLD and its progression to NASH (e.g., increased consumption of sugars, such as fructose [103]), but carefully controlled studies do not support this notion [104,105]. Overall, available studies highlight a key role for hepatic *de novo* fatty acid synthesis in IHTG accumulation; however the observation that increased *de novo* lipogenesis is not a uniform feature in all individuals with hepatic steatosis [106] reinforces the importance of other factors in the development and maintenance of excessive fat accumulation, including those related to liver fat mobilization.

### 5.2. Intrahepatic Fatty Acid Mobilization in NAFLD

#### 5.2.1. Hepatic Fatty Acid Oxidation and Ketogenesis

Oxidation of fatty acids provides the major source of energy for the liver; together with amino acid oxidation, it accounts for more than 90% of basal hepatic energy requirements [107]. Fatty acid oxidation occurs primarily in the mitochondria (*beta*-oxidation) and to a much lesser extent in peroxisomes and microsomes (mainly the initial steps of *beta*-oxidation of very long chain fatty acids, but also *alpha*-oxidation) [108]. Beta-oxidation requires fatty acids to be transported from the cytoplasm to the mitochondrial matrix, *i.e.*, across the mitochondrial double membrane. This process requires the “activation” of fatty acids by coenzyme A, which is accomplished by fatty acyl-CoA synthetase in the cytoplasm [109]. Fatty acyl-CoA is then converted to fatty acyl carnitine by carnitine palmitoyl transferase (CPT) 1 in the outer mitochondrial membrane, which is then shuttled across the inner mitochondrial membrane by carnitine translocase [109]. Finally, CPT 2 regenerates fatty acyl CoA and free carnitine inside the mitochondrial matrix [109]. *Beta*-oxidation consists of repetitive cycles of dehydrogenation, hydration, and cleavage reactions, progressively shortening the fatty acyl-CoA chain by two carbon units per cycle, which are released as acetyl-CoA [110]. Several membrane-bound and soluble enzymes are involved in this process, varying in acyl chain length specificity [110]. Under normal circumstances, mitochondrial *beta*-oxidation is quantitatively the major pathway of intracellular fatty acid oxidation [108].

Acetyl-CoA generated in the mitochondrial matrix through *beta*-oxidation enters the tricarboxylic acid cycle and the electron transport chain, where complete oxidation to carbon dioxide generates energy for the liver [111]. When oxidation of fatty acids proceeds at very high rates (e.g., during starvation), acetyl-CoA is produced in excess of the amount that can be disposed in the tricarboxylic acid cycle; under these conditions, two acetyl-CoA molecules condense to form acetoacetate, which can be converted to β-hydroxybutyrate or spontaneously turn into acetone (all of which are considered “ketone bodies”) [111]. Ketone bodies are then released into the bloodstream and provide a source of energy for extrahepatic tissues, including the skeletal muscle, heart, and brain [109,112,113]. Regulation of beta-oxidation flux can occur at many levels, but largely depends on the rate of delivery of fatty acids into the mitochondrial matrix, which in turn depends on cytoplasmic fatty acid availability and the activity of CPT1 [114]. A long-known inhibitor of CPT1 is malonyl-CoA that is produced by the carboxylation of acetyl-CoA during *de novo* lipogenesis [115]. Regulation of ketogenesis depends on all factors controlling *beta*-oxidation and the ratio of acetyl-CoA to free CoA in the mitochondrial matrix [111]. The two main pathways of intracellular fatty acid metabolism (*de novo* lipogenesis and *beta*-oxidation/ketogenesis) proceed at opposite directions under tight metabolic control, so that when one direction is favored, the other is inhibited.

Given that hepatic *de novo* lipogenesis is augmented in NAFLD [95,97,98], it is not unreasonable to expect that hepatic *beta*-oxidation/ketogenesis would be suppressed [116]. Data from studies conducted in rodent models demonstrate that modulation (up- or down-regulation) of intrahepatic fatty acid oxidation by a variety of means influences IHTG content. Genetic or experimentally-induced deficiency of enzymes involved in mitochondrial *beta*-oxidation leads to accumulation of IHTG [117,118], and vice versa [119,120,121,122,123]. Studies in human subjects, however, provide controversial results, and their interpretation is further complicated by the absence of a reliable method to directly measure fatty acid oxidation in the liver *in vivo*. Hepatic CPT1 gene expression is typically lower, but gene expression of several other key enzymes of hepatic fatty acid oxidation is actually greater in subjects with NAFLD than those with normal IHTG content [89,99,100,101]. Other indirect measures of fatty acid oxidation in the liver, such as plasma ketone body concentrations, suggest that hepatic fatty acid oxidation is either normal or increased in people with NAFLD [29,30,124,125]. Nevertheless, circulating ketone bodies are likely an unreliable marker of hepatic fatty acid oxidation, given the unstable chemical nature of ketone bodies (leading to their rapid interconversion and/or conversion to acetone) but also the fact that their plasma concentrations reflect the net balance between the rate of hepatic ketogenesis and the rates of extrahepatic ketone body utilization. Early tissue perfusion studies reported that hepatic ketogenesis could be an important determinant of IHTG accumulation, as it can dispose of as much as two-thirds of the fat entering the liver [126]. However, a recent study in an knockout rodent model demonstrated that impaired hepatic ketogenesis can be an important determinant of NAFLD progression to NASH, but not NAFLD development itself [127], and the only available study to date that measured rates of ketogenesis *in vivo* in human subjects, by means of β-hydroxybutyrate tracer dilution, found no differences between subjects with and without NAFLD, matched for BMI and percent body fat [84].

Interestingly, it has been reported that NAFLD is associated with hepatic mitochondrial structural and functional abnormalities, including loss of mitochondrial cristae and paracrystalline inclusions [29,128], decreased mitochondrial respiratory chain activity [129], impaired ability to resynthesize ATP following a fructose challenge [130], and increased hepatic uncoupling protein 2 [100]. All of these abnormalities could affect hepatic energy production but not fatty acid oxidation, and could represent an adaptive uncoupling of fatty acid oxidation and ATP production, which allows the liver to oxidize excessive fatty acid substrates without generating unneeded ATP. The summation of these observations likely refutes the decrease in hepatic fatty acid oxidation as a major contributor to IHTG accumulation and the pathogenesis of NAFLD in humans. In addition, data from animal studies and *in vitro* experiments suggest there are several rescue pathways of fatty acid oxidation in conditions of insufficient mitochondrial *beta*-oxidation, including peroxisomal fatty acid oxidation and *omega*-oxidation [108,131]. Very little—if anything—is known regarding these pathways of fatty acid utilization in NAFLD, although there are some reports of augmented expression of peroxisomal (*beta*-oxidation) and microsomal (*omega*-oxidation) enzymes of fatty acid oxidation in liver samples from patients with NAFLD than those without NAFLD [100].

#### 5.2.2. Hepatic VLDL-triglyceride Secretion

An important pathway of mobilization of liver fat consists of triglyceride export in the hydrophobic core of VLDL. Hepatic VLDL assembly occurs in two steps, both of which require the action of microsomal triglyceride transfer protein (MTP) [132]. The process involves the partial lipidation of a newly synthesized apolipoprotein B-100 molecule to form a small and dense VLDL precursor (first step), and the fusion of this small and dense precursor with a large triglyceride droplet to form a mature and triglyceride-rich VLDL (second step), which is subsequently secreted into plasma [132]. Each VLDL particle contains a single molecule of apolipoprotein B-100 [133], which is a large polypeptide that provides and maintains the structure of the whole lipoprotein particle and remains bound to the VLDL throughout its intravascular metabolism; whereas the availability of triglyceride in the core of nascent VLDL varies considerably (from ~5000 to ~50,000 in human subjects *in vivo*) and can determine some aspects of the metabolic fate of VLDL [134].

Fatty acids are made available to the liver from several sources. The major source of hepatic fatty acids is the systemic plasma FFA pool, which consists of fatty acids derived predominantly from subcutaneous adipose tissue lipolysis; fatty acids are made available to the liver from several nonsystemic sources as well, including hepatic *de novo* lipogenesis and lipolysis of triglyceride already stored in the liver (intrahepatic fat) and intra-abdominal adipose tissue (visceral fat), or carried back to the liver after lipoprotein uptake (e.g., LDL and HDL) [135]. In healthy postabsorptive subjects, the majority (65%–75%) of fatty acids utilized for hepatic VLDL-triglyceride secretion are derived from the systemic plasma FFA pool; a small portion (<5%) originates from hepatic *de novo* lipogenesis, with the remainder (20%–30%) likely originating from the lipolysis of intrahepatic, visceral, and circulating lipoprotein triglyceride [136,137]. Fatty acids that are not oxidized in the liver are esterified to triglyceride, which can either be stored within the hepatocyte or incorporated into VLDL and secreted into the circulation. The precise mechanisms controlling the channeling of fatty acids towards storage or secretion are not entirely clear, however their origin (e.g., synthesized *de novo*, or taken up from plasma, or derived from lipolysis of stored triglyceride) may be an important factor [138,139]. In any case, by providing a mechanism for triglyceride export, VLDL secretion could be a means to reduce IHTG accumulation. Conversely, impaired hepatic VLDL secretion caused by genetic defects (familial hypobetalipoproteinemia) [140], pharmacological agents that inhibit MTP [141] or VLDL sorting through the Golgi apparatus [142], or deletion of CD36 [143], is associated with increased IHTG content. Surprisingly, data from most [60,144] but not all [95] studies in human subjects indicate that NAFLD is associated with a marked increase in hepatic VLDL-triglyceride secretion rate, independent of BMI and percent body fat. The majority of this increase is accounted for by a marked increase in the secretion of VLDL-triglyceride from nonsystemic fatty acids, presumably derived from the lipolysis of intrahepatic and visceral fat and *de novo* lipogenesis [60]. These findings demonstrate that increased IHTG content results in oversecretion of VLDL-triglyceride; however the increase in hepatic VLDL-triglyceride secretion is not sufficient to prevent or reverse NAFLD. This notion is supported by the observation that hepatic VLDL-triglyceride secretion rate increases linearly with increasing IHTG content within the normal range (up to 5%–10% of liver volume), but plateaus thereafter with IHTG content within the NAFLD range (>10% of liver volume) [60].

The mechanism(s) responsible for the inadequate increase in hepatic VLDL-triglyceride export in NAFLD is not known. The secretion rate of VLDL-apolipoprotein B-100 from the liver, which directly reflects the secretion of VLDL particles themselves (since each VLDL particle contains one molecule of apolipoprotein B-100), is either not different [60] or only slightly greater [145] in subjects with NAFLD than those without NAFLD. Hence the molar ratio of VLDL-triglyceride secretion rate to VLDL-apolipoprotein B-100 secretion rate, which is an index of the triglyceride content (and therefore, of the size) of newly-secreted VLDL particles from the liver, is more than two-fold greater in people with NAFLD [60]. These results suggest the liver in NAFLD secretes triglyceride-richer and larger in size VLDL. Studies in rodents found that very large VLDL particles cannot be secreted from the liver because they exceed the diameter of the sinusoidal endothelial pores, and this inability can result in excessive IHTG accumulation and the development of steatosis [146]. On the contrary, pharmacological induction of apolipoprotein B-100 secretion can help reduce triglyceride accumulation in hepatocytes [147]. It therefore seems an imbalance between the secretion of VLDL-triglyceride and VLDL-apolipoprotein B-100, resulting from a “failure” to sufficiently upregulate hepatic VLDL-apolipoprotein B-100 secretion to match the excess VLDL-triglyceride secretion, is central in the development and maintenance of NAFLD associated with obesity. This is not the case when fatty liver occurs outside the context of obesity, e.g., in protein-energy malnutrition [148]. Apolipoprotein B-100 is typically synthesized in hepatocytes at rates that far exceed those required for VLDL secretion; in fact the majority of the protein produced is recycled intracellularly [149], suggesting that the rate of degradation likely controls the amount of apolipoprotein B-100 available for secretion. An increasing body of evidence suggests that hepatic ER stress, induced by the oversupply of fatty acids, augments intracellular proteolysis of apolipoprotein B-100 and thereby reduces the secretion of VLDL-apolipoprotein B-100 [147,150,151]. The metabolic inability to secrete more VLDL particles (to export more triglyceride in more particles) in conjunction with the physical inability to secrete larger and larger VLDL particles (to export more triglyceride per particle) are likely important contributing factors to excessive IHTG accumulation and potentially to the development of NAFLD.

**Figure 2:**
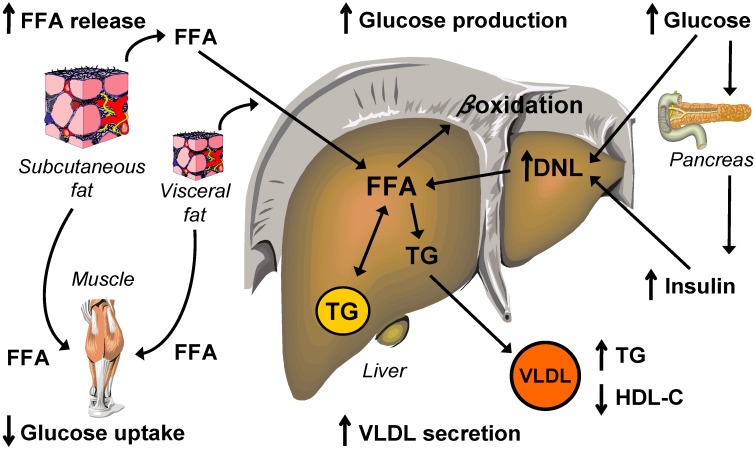
Mechanisms of metabolic dysfunction in NAFLD. Nonalcoholic fatty liver disease (NAFLD) is associated with increased release of free fatty acids (FFA) from adipose tissue, because of enlarged fat stores and the inability of insulin to down-regulate lipolysis (*i.e.*, adipose tissue insulin resistance); systemic plasma FFA are subsequently taken up by the liver at increased rates. Intrahepatic fatty acid availability is further augmented by increased hepatic *de novo* lipogenesis (DNL). Increased availability of fatty acids in the liver results in accumulation of intrahepatic triglyceride (TG) and steatosis, oversecretion of very low density lipoprotein (VLDL) TG and various form of dyslipidemia, and increased glucose production (*i.e.*, hepatic insulin resistance). Increased availability of fatty acids in muscle results in decreased glucose uptake (*i.e.*, skeletal muscle insulin resistance). The net result of increased glucose production and decreased glucose uptake is an increase in plasma glucose concentration (hyperglycemia), which stimulates the pancreas to secrete more insulin, to compensate for the impaired insulin action. Hyperglycemia and hyperinsulinemia further augment hepatic *de novo* lipogenesis.

## 6. Conclusions

Hepatic steatosis (*i.e.*, NAFLD) is a common feature of obesity and is associated with a plethora of metabolic abnormalities including: (1) multi-organ insulin resistance, reflected by the inability of insulin to downregulate hepatic glucose production (liver insulin resistance) and subcutaneous adipose tissue lipolysis (adipose tissue insulin resistance), and to stimulate muscle glucose uptake (skeletal muscle insulin resistance), (2) increased pancreatic β-cell insulin secretion to compensate for the impaired insulin action, (3) increased intrahepatic fatty acid availability from systemic (*i.e.*, plasma) and nonsystemic (e.g., *de novo* lipogenesis) sources, and (4) increased hepatic triglyceride export in the form of VLDL, which leads to various forms of dyslipidemia (Figure 2). Despite the progress that has occurred in recent years in the understanding much of the pathophysiology of NAFLD, it still remains unclear whether the presence of liver steatosis is a cause or a consequence of metabolic dysfunction. It is likely that both possibilities are true, so that different initiating events can lead to the same adverse metabolic spiral of excessive IHTG accumulation and deteriorated metabolic function. There is no doubt, however, that hepatic steatosis is a robust marker of metabolic dysfunction: increased IHTG content is a better marker of multi-organ insulin resistance and oversecretion of VLDL-triglyceride than whole-body or visceral adiposity [32,152], and predicts adverse metabolic changes induced by moderate weight gain [34]. A better characterization of the complex metabolic interactions leading to, or resulting from, hepatic steatosis will lead to the identification of novel therapeutic targets that can be manipulated by lifestyle (e.g., diet and exercise [153]) or pharmacological interventions to prevent or reverse hepatic steatosis and its unfavorable metabolic sequalae.
